# Effects of Preharvest Calcium Chloride and Postharvest UV‐B Irradiation and Cyanocobalamin Treatments on Storage Quality and Bioactive Properties of Beet Microgreens

**DOI:** 10.1002/fsn3.72137

**Published:** 2026-07-23

**Authors:** Zahra Torabi Mirabadi, Fariba Khalili, Elmira Ziya Motalebipour

**Affiliations:** ^1^ Department of Horticulture, Faculty of Agriculture, Water, Food and Nutraceuticals, Isf. C Islamic Azad University Isfahan Iran; ^2^ Medicinal Plant Research Center, Isf. C Islamic Azad University Isfahan Iran; ^3^ Department of Agronomy and Plant Breeding, Faculty of Agriculture, Water, Food and Nutraceuticals, Isf. C Islamic Azad University Isfahan Iran

**Keywords:** antioxidant capacity, beet microgreens, calcium chloride, cyanocobalamin, UV‐B irradiation

## Abstract

Microgreens are emerging functional foods valued for their unique color, intense flavor, and high levels of bioactive compounds, which contribute to human health. Despite their nutritional potential, commercial production and marketability are often limited due to rapid postharvest quality deterioration and inherently short shelf‐life. This study aimed to evaluate the effects of preharvest calcium chloride (CaCl_2_) application and postharvest treatments including UV‐B irradiation and cyanocobalamin on the photosynthetic pigments, bioactive compounds, antioxidant capacity, and visual quality of beet ( *Beta vulgaris* L.) microgreens during storage. Beet microgreens were cultivated under controlled environmental conditions and sprayed daily with CaCl_2_ at concentrations of 0, 1, and 10 mM for 14 days prior to harvest. After harvest, microgreens were treated with UV‐B irradiation at 0, 0.09, and 0.18 kJ m^−2^ s^−1^ or with cyanocobalamin at 0, 0.05, and 2 mM. Fresh‐cut microgreens were packaged in sealed polyethylene bags and stored at 4°C ± 1°C for four days. Key quality parameters, including total chlorophyll, visual quality, total phenolic content, antioxidant capacity, and reducing sugars, were measured during storage. Results indicated that beet microgreens were highly responsive to both preharvest and postharvest treatments, with effects strongly dependent on treatment concentration, UV‐B dose, and storage duration. Preharvest CaCl_2_ significantly increased pigment content, visual quality, antioxidant capacity, phenolic content, and sugar levels, with 10 mM yielding the highest values. Postharvest UV‐B further enhanced pigment retention, antioxidant activity, and phenolic accumulation, while low‐dose cyanocobalamin negatively affected all traits. Extended storage led to quality decline in all treatments, but UV‐B‐treated microgreens maintained the slowest rate of deterioration.

Overall, the combination of preharvest CaCl_2_ and postharvest UV‐B irradiation was the most effective strategy for preserving the nutritional, functional, and visual quality of beet microgreens. These findings demonstrate the importance of integrating preharvest nutritional management with targeted postharvest physical treatments to improve shelf‐life and commercial value.

## Graphical abstract

Preharvest calcium chloride application combined with postharvest UV‐B irradiation effectively enhances photosynthetic pigments, antioxidant capacity, and shelf‐life quality of beet microgreens during storage.

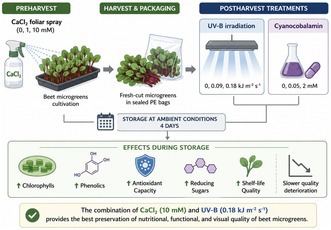

## 1 Introduction

The rapid growth of the global population, along with the accelerating expansion of urban areas, has created significant challenges in ensuring the availability of fresh, nutritious, and high‐quality food. These pressures have highlighted the need to adopt innovative food‐production systems capable of providing stable yields while operating within limited space and under variable environmental conditions. Controlled Environment Agriculture (CEA) has emerged as one of the most effective solutions to these challenges by enabling precise management of temperature, humidity, light, nutrients, and air composition to produce vegetables and microgreens regardless of outdoor conditions (Graamans et al. 2018; Zhang et al. 2021; Srimal et al. 2024).

Among CEA crops, microgreens have gained remarkable attention due to their short production cycle (7–21 days), minimal space requirements, low production costs, and exceptionally high nutritional value (Bhaswant et al. 2023; Teng et al. 2023). Microgreens are harvested shortly after the cotyledon expansion, typically at the early stage of true leaf development.

Despite their advantages, microgreens exhibit a notably short shelf life, often lasting less than one week after harvest due to their fragile tissues, high moisture content, and elevated metabolic activity (Kyriacou et al. 2019).

Originally introduced in the 1990s in high‐end Californian restaurants, microgreens have since become increasingly popular in households, retail markets, and the food industry (Lenzi et al. 2019). A wide range of species—such as radish, coriander, amaranth, sunflower, cress, broccoli, onion, and beet—are cultivated for microgreen production (Samotyja et al. 2012). Numerous studies have demonstrated that microgreens contain greater levels of vitamins, minerals, carotenoids, phenolic compounds, and antioxidants compared with their mature counterparts (Yadav et al. 2019; Puccinelli et al. 2019). This dense nutritional profile has led to microgreens being classified as “superfoods” (Bhaswant et al. 2023). However, their rapid deterioration postharvest—manifested by wilting, discoloration, decay, and loss of bioactive compounds—has become a major obstacle to their broader commercialization. Consequently, pre‐harvest and postharvest treatments are increasingly recognized as essential tools for enhancing quality, delaying senescence, and extending shelf‐life (Allegretta et al. 2019; Brahmakshatriya et al. 2022; Nair 2023).

Among the various strategies applied before harvest, calcium supplementation—particularly in the form of calcium chloride (CaCl_2_)—is widely acknowledged for its crucial role in maintaining membrane integrity, strengthening cell walls, reducing electrolyte leakage, and mitigating senescence (Kou et al. 2014; Lu et al. 2018). Calcium acts as a structural component of cell walls and contributes to stress tolerance by stabilizing pectin cross‐linking. Previous studies have shown that preharvest CaCl_2_ treatments in Brassica microgreens lead to increased biomass, reduced microbial growth, enhanced antioxidant enzyme activities, and elevated glucosinolate content (Yang et al. 2015; Lu et al. 2018). Nevertheless, research exploring the effects of CaCl_2_ on beet microgreens remains scarce.

Due to the extreme perishability of microgreens, effective postharvest treatments are vital for maintaining their marketability. UV‐B light is known to stimulate the biosynthesis of secondary metabolites by activating the UVR8 signaling pathway, which induces the accumulation of flavonoids, phenolics, and antioxidant compounds (Martínez‐Zamora et al. 2021). While UV‐B treatments have been explored in some Brassica microgreens, the postharvest response of beet microgreens to UV‐B remains largely unexplored.

Vitamin B_12_ contributes to essential metabolic processes in plants, including methionine synthesis and stress modulation. Exogenous cyanocobalamin application has been reported to enhance total phenolics, flavonoids, and ascorbic acid in certain fresh produce items (El‐Bary 2017; Supapvanich et al. 2020). Despite its potential, the effects of vitamin B_12_ on microgreens—particularly beet microgreens—have not yet been comprehensively investigated.

Beet ( *Beta vulgaris* L.) microgreens are prized for their intense color and their rich content of betalains, phenolic compounds, antioxidants, and anti‐inflammatory metabolites. Betalains have been associated with anti‐cancer, anti‐oxidative, and cytoprotective effects (Rocchetti et al. 2020). Given the high nutritional value of beet microgreens and their rapid perishability, applying effective pre‐ and postharvest treatments is essential for preserving quality, extending shelf‐life, and enhancing bioactive compounds. Although the use of calcium chloride as a preharvest treatment and postharvest methods such as UV‐B irradiation and cyanocobalamin application have been investigated in some fresh produce, available information regarding the effects of these three treatments on beet microgreens is very limited, and no comprehensive study has yet evaluated all of these interventions simultaneously.

Therefore, this research was conducted to examine the effects of preharvest calcium chloride (CaCl_2_) application and postharvest treatments including UV‐B irradiation and cyanocobalamin (vitamin B_12_) on the physical, chemical, nutritional, and functional characteristics of beet microgreens during storage. Specifically, the study aims to assess changes in visual quality, antioxidant activity, chlorophylls, phenolic compounds, as well as to explore potential effects among these treatments. The ultimate goal of this research is to provide optimized strategies for extending shelf‐life and preserving the nutritional quality of beet microgreens within controlled environment agriculture systems and fresh‐produce supply chains.

## 2 Materials and Methods

### 2.1 Experimental Site

**FIGURE 1:**
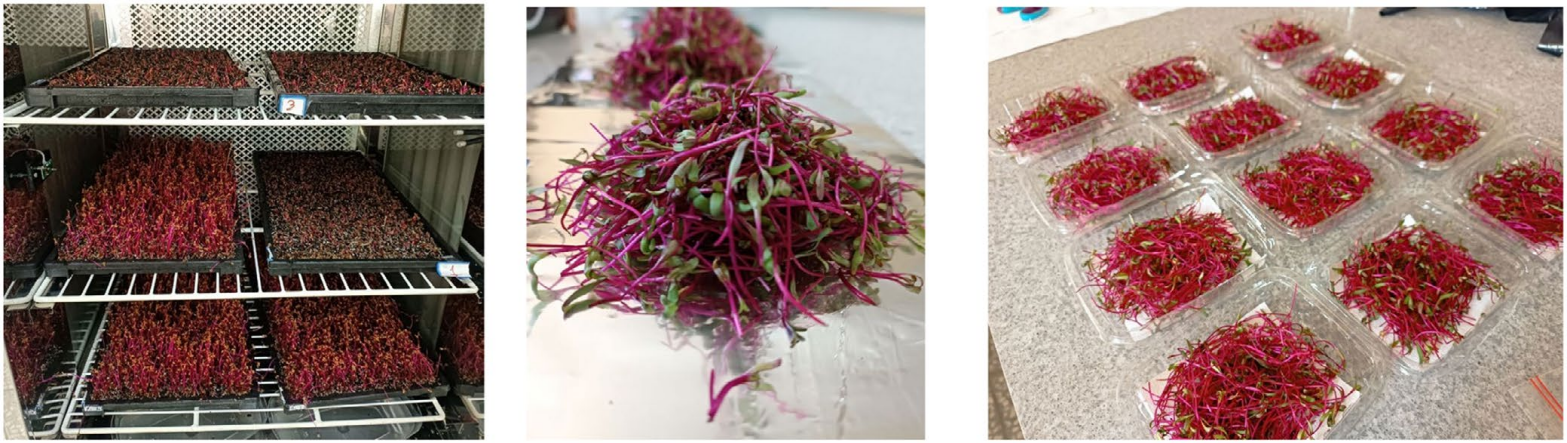
Overview of the cultivation, harvesting, and packaging processes of the studied beet microgreens.

The experiment was conducted under controlled laboratory conditions at the Medicinal Plant Rsearch Center, Islamic Azad University (Isfahan, Iran), over a 6‐month period in 2023. Microgreens were grown in pre‐sanitized plastic trays (30 × 60 × 5 cm). Figure 1 illustrates the growth facility and seedling trays used in the experiment.

### 2.2 Plant Material, Experimental Design, and Procedure

High‐quality, disease‐free beet ( *Beta vulgaris* L.) seeds were manually selected and surface‐sterilized through sequential washing with distilled water, treatment with a mild detergent solution, and thorough rinsing with sterile distilled water.

Seed disinfection was performed using a thermal shock method, in which seeds were immersed in hot water at 75°C for 20 s followed by immersion in cold water at 0°C for 20 s. This cycle was repeated five times. After thermal treatment, seeds were soaked in sterile distilled water for 12 h at 25°C and subsequently pre‐germinated on moist sterile pads for 24 h.

Pre‐germinated seeds were sown in pre‐sterilized trays containing a uniform growth substrate consisting of a 1:1 mixture of perlite and cocopeat. Trays were maintained in a controlled germination chamber at 25°C and 60% relative humidity. Germination was carried out in complete darkness for 7 days. Thereafter, seedlings were exposed to a 16 h light / 8 h dark photoperiod under controlled growth conditions.

To resolve inconsistencies in harvest timing, all microgreens were harvested at a uniform growth stage of 14 days after sowing by cutting just above the substrate surface.

Preharvest calcium chloride (CaCl_2_) treatments were applied as foliar sprays at three concentrations (0, 1, and 10 mM) throughout the 14‐day growth period under light conditions. Following harvest, microgreens from each CaCl_2_ treatment were divided into two postharvest treatment groups to form a factorial experimental design.

One group was subjected to UV‐B irradiation, while the other was treated with cyanocobalamin.

For postharvest cyanocobalamin treatment, harvested microgreens were immersed in vitamin B_12_ solutions at concentrations of 0, 0.05, and 2 mM for 5 min, followed by gentle surface drying under sterile conditions.

For postharvest UV‐B treatment, freshly harvested beet microgreens were immediately exposed to UV‐B irradiation at three levels: 0 (control under normal light), 0.09, and 0.18 kJ m^−2^. Irradiation was performed at room temperature. UV‐B exposure was applied using two UV‐B lamps (TUV G30T8, 30 W, Philips, Amsterdam, The Netherlands). The peak emission wavelength of the lamps was in the UV‐B range (approximately 280–315 nm). Samples were placed at a fixed distance of 40 cm from the light source. The irradiance intensity was measured and calibrated using a UV radiometer prior to treatment to ensure accurate dose delivery. Exposure time was adjusted according to the required radiation dose (0, 0.09, and 0.18 kJ m^−2^). After irradiation, all samples were immediately transferred to storage at 4°C ± 1°C.

Following the treatments, 30 g portions of microgreens were packaged in polyethylene bags (25 μm thickness) and sealed at the top. The samples were stored at 4°C ± 1°C with 90% ± 5% relative humidity and their physiological parameters were evaluated on a daily basis throughout the storage period (4 days). Samples were immediately frozen in liquid nitrogen and stored at −70°C until analysis.

### 2.3 Sensory Evaluation

The visual quality of minimally processed beet microgreens was evaluated by a trained sensory panel consisting of 10 assessors (5 females and 5 males). Prior to evaluation, panelists received training sessions to familiarize them with the evaluation scale, quality attributes, and scoring procedure. Samples were presented in a randomized order under controlled laboratory conditions (25°C ± 2°C, neutral lighting, and odor‐free environment) to minimize external bias. Evaluations were conducted at each storage interval throughout the shelf‐life period.

Appearance and off‐odor were rated using a five‐point hedonic scale, where 1 = extremely poor, 3 = limit of marketability, and 5 = excellent quality (Lopez‐Rubira et al. 2005). Each sample was evaluated independently, and panelists were instructed to cleanse their palate between samples.

### 2.4 Chlorophyll

Photosynthetic pigments were determined according to Lichtenthaler and Buschmann (2001) with minor modifications. Fresh leaf tissue (0.1 g) was homogenized in liquid nitrogen and extracted in 10 mL of cold organic solvent consisting of methanol:acetone (1:2 v/v). The mixture was shaken in darkness for 4 h at 4°C to prevent pigment degradation. The extract was then centrifuged and phase separation was achieved by adding 1 mL of 1 M NaCl solution. The supernatant was collected for spectrophotometric analysis. Absorbance was measured using a UV–Vis spectrophotometer (model specified if available) at 644 nm and 662 nm. Chlorophyll contents were expressed on a fresh weight basis (mg kg^−1^ FW). Chlorophyll a, chlorophyll b, and total chlorophyll were calculated using the following equations and all measurements were performed in triplicate.

Chla=10.05A662–0.766A644

Chlb=16.37A644–3.14A662

Total chlorophyll=Chla+Chlb

### 2.5 Total Phenolic Content

Total phenolic content was determined using the Folin–Ciocalteu colorimetric method according to Singleton and Rossi (1965) with slight modifications. Briefly, 0.5 g of frozen and finely ground tissue was homogenized with 3 mL of methanol–water (70:30, v/v). The mixture was extracted for 60 min on an orbital shaker at 3000 rpm under dark conditions while kept on ice. Smples were then centrifuged at 15,000 × g, 10 min at 4°C, and the clear supernatant was collected for analysis.

An aliquot (19.2 μL) of the extract was mixed with 29 μL of 1 N Folin–Ciocalteu reagent. After a 3‐min incubation in darkness at room temperature, 192 μL of a solution containing 0.4% sodium carbonate and 2% sodium hydroxide was added. The reaction mixture was incubated for 1 h, and absorbance was measured at 750 nm using a microplate spectrophotometer.

Total phenolic content was quantified from a gallic acid calibration curve and expressed as mg gallic acid equivalents (GAE) per g fresh weight. All measurements were performed in triplicate.

### 2.6 Antioxidant Capacity

The radical‐scavenging activity of microgreens was assessed using the DPPH assay based on the procedure described by Brand‐Williams et al. (1995), with slight modifications. A methanolic DPPH solution (0.7 mM) was prepared 2 h prior to analysis, and its absorbance was adjusted to 1.1 ± 0.2 at the time of use.

For each determination, 21 μL of extract was combined with 194 μL of the DPPH working solution and allowed to react for 30 min at room temperature under dark conditions. The reduction in absorbance was measured at 515 nm using a microplate spectrophotometer. Antioxidant activity was calculated from an ascorbic acid standard curve and expressed as ascorbic acid equivalent antioxidant capacity (AEAC). All samples were analyzed in triplicate.

### 2.7 Reduced Sugars

Reducing sugars were quantified using the Somogyi–Nelson method (Nelson 1944). Briefly, 2 g of frozen, ground microgreens were extracted with 12 mL of ethanol by homogenization. The homogenate was centrifuged at 12,000 × g for 15 min at 4°C, and the resulting supernatant was collected for analysis.

The concentration of reducing sugars was determined colorimetrically by measuring absorbance at 500 nm. Quantification was carried out using a calibration curve prepared with glucose as the standard.

### 2.8 Statistical Analysis

Data were initially organized and tabulated using Microsoft Excel and assessed for normality using the Kolmogorov–Smirnov test (SPSS software). The experiment was arranged as a factorial experiment within a completely randomized design (CRD) with three replicates per treatment. Statistical analyses were performed using SAS software (SAS Institute Inc., Cary, NC, USA). Analysis of variance (ANOVA) was conducted based on the factorial model to evaluate the main effects of preharvest calcium chloride concentrations, postharvest UV‐B irradiation, cyanocobalamin treatments, and their interaction effects. When significant differences were detected, means were separated using Duncan's Multiple Range Test (DMRT) at a significance level of *p* ≤ 0.05. Graphical illustrations were prepared using Microsoft Excel.

## 3 Results and Discussion

### 3.1 Visual Quality

**FIGURE 2:**
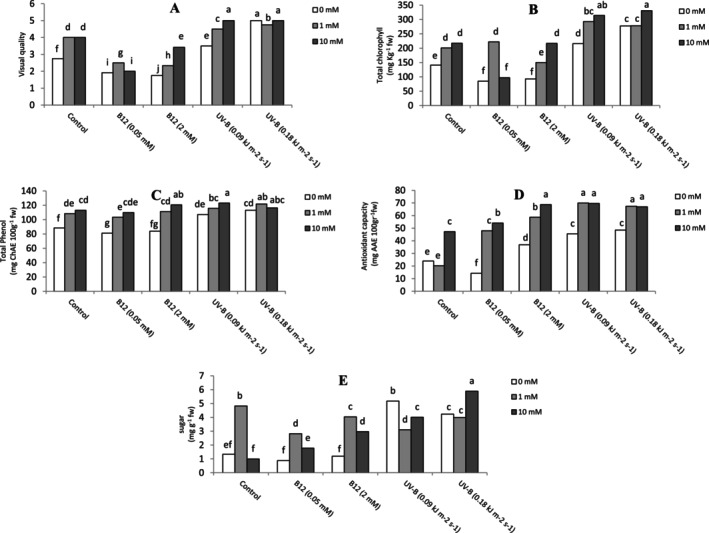
Comparison of the mean interaction effects of pre‐harvest calcium chloride treatment and post‐harvest treatments (A) Visual quality, (B) Total chlorophyll, (C) Total phenol, (D) Antioxidant capacity, (E) Sugar in beet microgreens.

**FIGURE 3:**
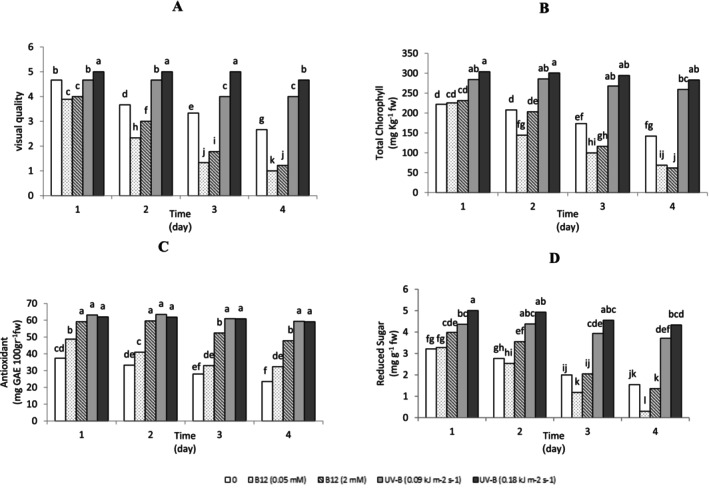
Comparison of the mean interaction effects of post‐harvest treatments (A: Visual quality, B: Total Chlorophyll, C: Antioxidant, D: Sugar) and time in beet microgreens.

Visual quality was significantly affected by preharvest CaCl_2_ application, postharvest treatments, and storage duration (*p* < 0.01), with significant interaction effects among these factors (Table 1). Microgreens subjected to pre‐harvest calcium chloride treatment at concentrations of 1 and 10 mM, respectively, in combination with UV‐B irradiation at the studied levels, showed the best visual quality (Figure 2). UV‐B treated microgreens maintained significantly greater visual quality scores than the control and other postharvest treatments during the entire storage period (*p* < 0.05). Moreover, higher UV‐B doses were associated with better preservation of visual quality, indicating a dose‐dependent delay in senescence (Figure 3).

**TABLE 1:** Mean squares of photosynthetic pigments for beet microgreens.

Sources of variation	Mean squares					
	Degrees of freedom	Total chlorophyll	visual quality	Antioxidant capacity	Total phenol	Reducing sugars
A	2	9742.47**	12.82**	11897.8**	8005.7**	21.32**
B	4	188121.58**	50.04**	7053.4**	2373.0**	52.51**
C	3	71943.90**	26.06**	872.1**	704.5**	28.21**
A × B	8	20115.63**	2.61**	781.9**	533.9**	23.60**
A × C	6	849.43^ns^	0.45**	78.1**	77.9^ns^	0.57*
B × C	12	9577.75**	2.71**	83.8**	64.0^ns^	2.15**
A × B × C	24	1422.68**	0.27**	32.6^ns^	16.5^ns^	0.45**
Total error	120	688.29	0.016	25.05	36.94	0.2273
C.V. %	—	12.56	3/69	10/14	5.63	15.15

*Note:* A: Pre‐harvest calcium chloride treatment, B: Post‐harvest treatment, C: Time and C.V.: Coefficient of variation ** and * and ns represent statistical significance at the 1% and 5% probability levels, and non‐significant, respectively.

The beneficial effects of calcium are primarily attributed to its ability to cross‐link pectic polysaccharides in the cell wall, stabilize cellular membranes, and reduce electrolyte leakage. These structural improvements slow physiological deterioration and extend postharvest longevity. Similar enhancements in visual quality following calcium application have been reported in leafy vegetables and microgreens (Bergquist et al. 2006; Pinto et al. 2015).

Postharvest UV‐B irradiation further improved visual quality, as treated microgreens showed delayed wilting, reduced yellowing, and higher visual acceptability throughout the storage period. The maintenance of quality under UV‐B treatment is likely associated with the induction of antioxidant defense systems and increased accumulation of phenolic compounds, which suppress senescence‐related processes (Sun et al. 2013).

In contrast, cyanocobalamin treatments negatively affected visual quality, particularly at low concentration. These samples exhibited accelerated yellowing, tissue breakdown, and rapid loss of freshness during storage. This decline in quality was consistent with the observed reductions in chlorophyll content and antioxidant capacity, suggesting that low‐dose cyanocobalamin may disrupt metabolic homeostasis and accelerate senescence processes (Figure 2).

### 3.2 Total Chlorophyll Content

Analysis of variance demonstrated that preharvest calcium chloride (CaCl_2_), postharvest treatments (UV‐B irradiation and cyanocobalamin), and storage time exerted highly significant effects (*p* < 0.01) on total chlorophyll content of beet microgreens (Table 1).

Chlorophyll responses were strongly influenced by the combined effects of preharvest and postharvest factors during storage (Figure 2). Pre‐harvest treatment with CaCl_2_, especially when combined with post‐harvest UV‐B irradiation, showed a significant effect on preserving chlorophyll and preventing its decline during the post‐harvest period. This treatment at a concentration of 10 mM, together with UV‐B irradiation at the two studied levels, exhibited the highest effectiveness in preventing chlorophyll degradation (Figure 2).

This enhancement can be attributed to the role of calcium in stabilizing chloroplast membranes, maintaining thylakoid integrity, and reducing membrane permeability, which collectively delay pigment degradation and senescence. Similar protective effects of calcium on chlorophyll stability have been reported in leafy vegetables and microgreens, where improved membrane integrity effectively slows senescence‐related pigment loss (Bergquist et al. 2006; Pinto et al. 2015; Abe and Oshita 2022).

Postharvest UV‐B irradiation further enhanced chlorophyll retention in beet microgreens. Both UV‐B doses resulted in higher chlorophyll levels compared with untreated samples, whereas cyanocobalamin—particularly at low concentration—caused a pronounced reduction in chlorophyll content. The decline observed under cyanocobalamin treatment may be associated with metabolic imbalance and enhanced oxidative stress, which accelerate chlorophyll catabolism. In contrast, UV‐B‐treated microgreens maintained chlorophyll stability throughout the storage period, confirming the protective role of UV‐B against postharvest senescence, as previously reported in microgreens and other leafy vegetables (Sun et al. 2013).

Although storage time generally led to progressive pigment loss, the rate of degradation varied markedly among treatments. Microgreens exposed to UV‐B exhibited minimal reductions in chlorophyll during storage, whereas untreated and cyanocobalamin‐treated samples showed rapid pigment decline. These results indicate that UV‐B irradiation can effectively mitigate storage‐induced chlorophyll degradation, particularly when combined with preharvest calcium nutrition (Figure 3).

### 3.3 Total Phenolic Content and Antioxidant Capacity

Preharvest CaCl_2_ and postharvest UV‐B irradiation significantly influenced total phenolic content and antioxidant capacity of beet microgreens (Table 1). Both calcium concentrations enhanced total phenolic content and antioxidant capacity, reflecting improved cellular stability and reduced oxidative damage. Calcium‐mediated reinforcement of membrane integrity likely limits the accumulation of reactive oxygen species, thereby preserving antioxidant potential (Figure 2).

UV‐B irradiation exerted a pronounced stimulatory effect on antioxidant capacity and phenolic accumulation (Figure 2). UV‐B‐treated microgreens consistently exhibited higher antioxidant activity and maintained these levels throughout storage (Figure 3). This response can be attributed to UV‐B‐induced activation of phenylpropanoid metabolism, resulting in enhanced synthesis of phenolic compounds that function as protective antioxidants. Similar UV‐B‐mediated increases in phenolics and antioxidant activity have been reported in Brassica and other microgreen species (Sun et al. 2013; Alrifai et al. 2021).

Cyanocobalamin showed a dose‐dependent effect on bioactive compounds. In microgreens treated with calcium chloride at a concentration of 10 mM, post‐harvest application of cyanocobalamin at the highest dose (2 mM) enhanced the content of phenolic compounds and the antioxidant defense mechanisms to the highest levels (Figures 2 and 3).

Low concentration caused significant reductions in antioxidant capacity and phenols. This contrasting response suggests that low‐dose cyanocobalamin may induce metabolic stress without activating sufficient protective mechanisms, whereas higher doses may trigger mild stress responses that partially stimulate secondary metabolism (Figure 2).

### 3.4 Soluble Sugars

Reducing sugars were significantly influenced by both preharvest calcium chloride (CaCl_2_) application and postharvest UV‐B irradiation (*p* < 0.05). UV‐B treatment at both applied doses (0.09 and 0.18 kJ m^−2^) effectively prevented the degradation of reducing sugars during storage and maintained significantly higher sugar levels compared with the control and other postharvest treatments (Figures 2 and 3). Moreover, increasing UV‐B dose was positively associated with enhanced sugar retention, indicating a dose‐dependent delay in carbohydrate degradation. Similar UV‐B–mediated preservation of soluble sugars has been reported in leafy vegetables and microgreens, where UV‐B exposure stimulated stress‐adaptive metabolic responses and reduced postharvest carbohydrate breakdown (Gui et al. 2018).

Preharvest foliar application of CaCl_2_ further contributed to the retention of reducing sugars. Microgreens treated with 10 mM CaCl_2_ during the growth period exhibited higher sugar content at harvest and throughout storage compared with untreated plants. The highest reducing sugar levels were consistently observed in microgreens receiving the combined treatment of 10 mM CaCl_2_ and postharvest UV‐B irradiation at 0.18 kJ m^−2^, suggesting a synergistic interaction between calcium nutrition and UV‐B exposure. Calcium is known to enhance membrane stability, reduce electrolyte leakage, and maintain cellular integrity, thereby limiting metabolic deterioration and sugar loss during storage (El Habbasha and Ibrahim 2015).

The synergistic effect of CaCl_2_ and UV‐B likely reflects complementary physiological mechanisms: calcium strengthens structural stability, while UV‐B induces protective metabolic adjustments that delay senescence‐related carbohydrate degradation. Nevertheless, it is important to consider that excessive UV‐B exposure may negatively affect growth performance or biomass accumulation, as reported in some microgreen species (Santin et al. 2022; Dou et al. 2019).

Overall, the integration of preharvest calcium nutrition with controlled postharvest UV‐B irradiation proved highly effective in preserving reducing sugars and maintaining carbohydrate stability during storage, thereby contributing to improved postharvest quality of beet microgreens.

### 3.5 Overall Treatment Performance

A comparative evaluation of treatments demonstrated that the combined application of 10 mM CaCl_2_ and UV‐B irradiation (0.18 kJ m^−2^ s^−1^) provided the best overall performance across all measured parameters, including chlorophyll retention, shelf‐life quality, antioxidant capacity, total phenolic content, and reducing sugars (Table 2). This integrated treatment not only maximized pigment stability and visual acceptability but also enhanced the accumulation and preservation of bioactive compounds throughout storage. The observed synergistic effect underscores the importance of combining preharvest nutritional management with targeted postharvest physical treatments to optimize both quality and functional value of beet microgreens (Artes‐Hernandez et al. 2022; Appolloni et al. 2022).

**TABLE 2:** Comparative evaluation of preharvest and postharvest treatments on beet microgreens quality and shelf‐life.

Trait	Best treatment combination	Effect	Storage stability
Visual quality	10 mM CaCl_2_ + UV‐B	Highest visual quality, firmness, and freshness	Maintained superior quality over 4 days
Total chlorophyll	10 mM CaCl_2_ + UV‐B 0.18 kJ m^−2^	Highest chlorophyll retention	Most stable pigment levels during storage
Total phenolic content	10 mM CaCl_2_ + UV‐B 0.18 kJ m^−2^	Highest phenolic accumulation	Better retention compared to control
Antioxidant capacity	10 mM CaCl_2_ + UV‐B 0.18 kJ m^−2^	Maximum antioxidant activity	Slowest decline during storage
Soluble sugars	10 mM CaCl_2_ + UV‐B 0.18 kJ m^−2^	Highest sugar content	Minimal reduction over storage

*Note:* LSD test at 5% probability; shared letters indicate no significant difference among means.

Overall, the findings indicate that preharvest CaCl_2_ application strengthens physiological stability by maintaining membrane integrity and delaying senescence‐related degradation processes. Meanwhile, postharvest UV‐B irradiation effectively stimulates phenolic metabolism and antioxidant defense mechanisms, thereby preserving pigments, bioactive compounds, and visual quality during storage. In contrast, cyanocobalamin—particularly at low concentration (0.05 mM)—negatively affected chlorophyll content, antioxidant capacity, phenolic accumulation, and overall shelf‐life quality, leading to accelerated deterioration and reduced marketability. These results highlight the effectiveness of integrating calcium nutrition before harvest with controlled UV‐B exposure after harvest as a practical strategy to extend shelf‐life and enhance the nutritional and functional quality of beet microgreens.

## 4 Conclusion

The present study demonstrated that both preharvest and postharvest treatments play a crucial role in preserving the physiological quality, bioactive compounds, and shelf‐life of beet microgreens. Preharvest application of calcium chloride significantly enhanced chlorophyll content, antioxidant capacity, reducing sugars, and overall visual quality, highlighting the importance of calcium nutrition in improving cellular stability and delaying senescence processes.

Postharvest UV‐B irradiation proved to be an effective strategy for maintaining chlorophyll pigments, enhancing phenolic accumulation, and preserving antioxidant capacity during storage. UV‐B‐treated microgreens exhibited slower quality deterioration, reduced pigment degradation, and improved visual acceptability compared with untreated samples. In contrast, cyanocobalamin—particularly at low concentration—had adverse effects on pigment stability, antioxidant capacity, and visual quality, leading to accelerated senescence and reduced marketability.

Notably, the combined application of 10 mM CaCl_2_ and UV‐B irradiation showed a synergistic effect, resulting in the highest retention of photosynthetic pigments, superior visual quality, and enhanced functional properties throughout storage. These findings emphasize the importance of integrating preharvest nutritional management with targeted postharvest treatments to optimize both quality and nutritional value of beet microgreens.

Overall, the results provide practical and applicable insights for the microgreen industry, suggesting that the strategic use of calcium fertilization before harvest and UV‐B irradiation after harvest can effectively extend shelf‐life and enhance the nutritional and functional quality of beet microgreens. This integrated approach may contribute to improved postharvest management practices and increased consumer acceptance of fresh microgreens.

## Author Contributions

**Elmira Ziya Motalebipour:** writing – original draft, writing – review and editing, software, resources, investigation. **Fariba Khalili:** conceptualization, writing – original draft, methodology, validation, supervision, resources, data curation, project administration. **Zahra Torabi Mirabadi:** funding acquisition, investigation, visualization, formal analysis.

## Funding

The authors have nothing to report.

## Disclosure

*Author Approval and Responsibility Statement:* All authors have read and approved the final version of the manuscript. F.K. (Fariba Khalili), as the corresponding author, had full access to all of the data in this study and takes complete responsibility for the integrity of the data and the accuracy of the data analysis.

## Ethics Statement

This study involved plant material (beet microgreens) and did not include any endangered or protected species. No human or animal subjects were used in the experimental design. Therefore, ethics approval was not required for the cultivation and postharvest experiments conducted in this study. However, all experimental procedures were performed in accordance with relevant institutional and international guidelines for good research practice.

## Consent

For the sensory evaluation component, informed consent was obtained from all panel participants prior to participation, and all procedures were conducted in compliance with general ethics standards for human sensory testing.

## Conflicts of Interest

The authors declare no conflicts of interest.

## Data Availability

The data supporting the findings of this study are available within the article. Additional data are available from the corresponding author upon reasonable request. Corresponding Author had full access to all of the data in this study and takes complete responsibility for the integrity of the data and the accuracy of the data analysis.
